# Longitudinal and Multimodal Recording System to Capture Real-World Patient-Clinician Conversations for AI and Encounter Research: Protocol for an Observational Study

**DOI:** 10.2196/84688

**Published:** 2026-03-24

**Authors:** Misk Al Zahidy, Kerly Guevara Maldonado, Luis Vilatuna Andrango, Ana Cristina Proano, Ana Gabriela Claros, Maria Lizarazo Jimenez, Esteban Gomez-Alvarez, David Toro-Tobon, Victor M Montori, Oscar J Ponce-Ponte, Juan P Brito

**Affiliations:** 1 Care and AI Laboratory, Knowledge and Evaluation Research Unit, Mayo Clinic, Rochester, MN, United States; 2 Division of Endocrinology, Diabetes, Metabolism, and Nutrition, Department of Medicine, Mayo Clinic, 200 First Street SW, Rochester, MN, 55902, United States, 1 507-206-5737; 3 Derriford Hospital, University Hospitals Plymouth NHS Trust, Plymouth, United Kingdom

**Keywords:** artificial intelligence, AI, digital health, electronic health records, endocrinology, feasibility studies, multimodal data capture, patient-clinician communication

## Abstract

**Background:**

The promise of artificial intelligence (AI) in medicine depends on its ability to learn from data that reflect what matters to patients and clinicians in the care process. Most existing models are trained on electronic health records (EHRs), which primarily capture biological measures but rarely the interactions and relationships between patients and clinicians. These relationships, central to how care is understood, negotiated, and delivered, unfold across multiple modalities, including voice, text, and video, yet remain largely absent from current datasets. As a result, AI systems trained solely on EHRs risk perpetuating a narrow biomedical view of medicine and overlooking the lived exchanges that define clinical encounters.

**Objective:**

This study aims to design, implement, and evaluate the feasibility of a longitudinal, multimodal system for capturing patient-clinician encounters, linking 360° video or audio recordings with postvisit surveys and EHR data, to create a foundational dataset for downstream AI research.

**Methods:**

This single-site study was conducted in an academic outpatient specialty clinic (Division of Endocrinology, Mayo Clinic, Rochester, Minnesota, United States). Adult patients attending in-person visits with participating clinicians were invited to enroll. Encounters were recorded using a 360° 2D monocular video camera and dual-channel audio. After each visit, patients completed a brief survey assessing relational empathy, satisfaction, visit pace, and treatment burden. Demographic and clinical data were extracted from the EHR. Feasibility was assessed using 5 prespecified end points: clinician consent, patient consent, recording success, survey completion, and data linkage across modalities.

**Results:**

Recruitment began in January 2025. By August 2025, 35 of 36 (97%) eligible clinicians and 212 of 281 (75%) approached eligible patients had consented. Of the consented encounters, 162 (76%) resulted in a complete 360° video recording, and the postvisit surveys were completed for 204 of 212 (96%) consented encounters, reflecting 1 survey per encounter. Data collection is ongoing as of December 2025, and further analyses will be reported in subsequent publications.

**Conclusions:**

This protocol describes a longitudinal multimodal encounter capture system that links 360° audio or video with postvisit surveys and EHR data. The study specifies operational definitions, workflows, feasibility end points, and governance procedures to support implementation and replication in other clinical settings.

## Introduction

The patient-clinician encounter is the central space where medicine is practiced, where problems are explored, treatment options are negotiated, and care plans are co-created. These encounters require attention to both biology and biography, encompassing not only laboratory results or physical findings but also the lived experience, values, and goals of patients. The quality of this interaction—how clinicians and patients listen, respond, and work together—determines whether care fits the realities of patients’ lives and enables them to flourish despite illness. As the primary site where medicine unfolds, encounters should also be the primary lens through which we study, evaluate, and improve care [1,2].

The current paradigm in medical artificial intelligence (AI) is shifting from unimodal models, which analyze a single data type, to multimodal systems that integrate diverse inputs such as images, text, and structured data. To contribute meaningfully to patient care, these systems must be trained on data that reflect clinical encounters rather than relying solely on electronic health records (EHRs). Clinical notes, while indispensable for documentation, are written largely for billing and regulatory purposes and, as a result, emphasize biological measures while neglecting relational and contextual aspects of care [3,4]. As a result, they are a poor surrogate for the lived dynamics of encounters and limit the capacity of AI systems to develop a holistic understanding of patients that is necessary to foster patient-centered care [5].

To capture both the biology and the biography of patients, including their experiences, values, and goals of care, future AI models must be trained on encounter data where communication unfolds across multiple modalities.

The primary bottleneck for developing person-centered AI is the lack of high-quality, real-world, longitudinal multimodal data from clinical encounters. No scalable process currently exists to record and integrate this information at the level needed to support AI development and research. Recording real-world visits may disrupt clinical workflows, and both patients and clinicians must be comfortable with how data are captured, stored, and used. Consent processes need to address not only privacy and confidentiality but also future applications in AI research. Institutions must ensure ethical oversight, secure storage, and clarity around ownership and secondary use. With little precedent for routine, large-scale capture of encounter data, a feasibility study is essential to determine whether it can be done in a way that is acceptable, minimally disruptive, and ethically robust [6,7].

To address this gap, we designed a feasibility study to test whether multimodal data from real-world patient-clinician encounters can be captured in a way that is practical, acceptable, and ethically sound. This protocol outlines the development of a replicable system that integrates 360° video and audio recordings with patient-reported surveys and EHR data. By focusing on feasibility end points, including clinician and patient consent, recording success, survey completion, and data linkage, this study aims to establish the foundation for the scalable collection of encounter data. Demonstrating feasibility is a critical step toward creating the longitudinal, multimodal datasets needed to train and validate next-generation AI models that reflect the complexity of clinical care.

## Methods

### Study Setting and Design

This protocol describes a step-by-step methodology to design and implement a longitudinal, multimodal encounter capture system in an academic outpatient specialty clinic (Division of Endocrinology, Mayo Clinic, Rochester, Minnesota, United States). This study is a prospective feasibility implementation protocol designed to evaluate whether a standardized multimodal encounter capture system can be integrated into routine outpatient care and assessed using predefined feasibility and acceptability end points. The protocol specifies the procedures, end points, and analysis plan for assessing the implementation of the encounter capture system.

Endocrinology was chosen because visits often involve chronic conditions that require longitudinal management, complex decision-making, and sustained patient-clinician collaboration. The investigators’ affiliation with the division further facilitated early approvals and integration with practice. However, this protocol can be implemented across multiple health care services.

### System Development

#### Multidisciplinary Design Process

A multidisciplinary team, including encounter researchers, endocrinologists, implementation scientists, AI researchers, and health-services researchers, uses a rapid, iterative design approach to develop a system capable of capturing rich, multimodal data from routine clinical encounters and to define standardized workflow and data management procedures.

#### Core Data Components

The system captures information from three integrated sources: (1) multimodal video recordings of clinical encounters; (2) postencounter patient surveys assessing satisfaction, relational empathy, and burden; and (3) structured and unstructured EHR data, including clinical notes, patient portal messages, and clinical variables.

### Participant Recruitment and Eligibility Criteria

#### Clinicians

All practicing clinicians in the Division of Endocrinology, including physicians, advanced practice providers, fellows, certified diabetes care and education specialists, and registered nurses, are eligible to participate. Clinicians are approached in person or via email, provided with detailed study information, and consented electronically on a tablet. Upon consent, each clinician is assigned a unique participant ID (eg, ENDOC###) and receives an email to complete a short demographic survey which includes their professional role, age, gender, race or ethnicity, and years in practice.

#### Patients

Patients are eligible if they are adults (aged ≥18 y), have a scheduled in-person appointment with a participating clinician, and can provide informed consent in English. Exclusion criteria include the presence of cognitive impairment, the need for an interpreter, or not being the primary individual responsible for managing their care. Enrolled patients are assigned a unique participant ID (ie, ENDOP###) for longitudinal linkage across visits.

**Figure 1.**
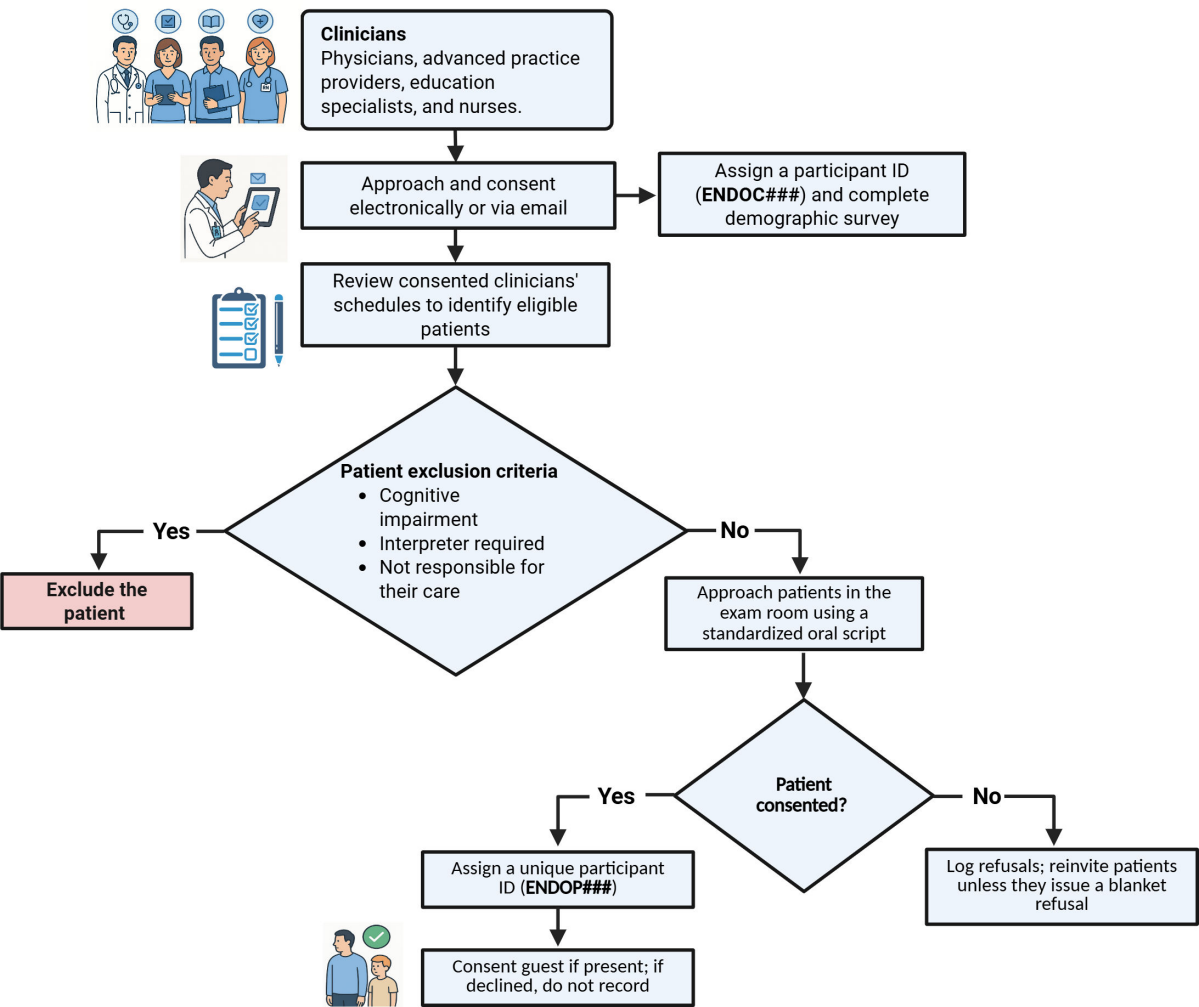
Recruitment and consent workflow for clinicians and patients.

Any adult guests present in the exam room (eg, spouse and adult child) must provide verbal consent before recording. If a guest declines to be recorded or if a minor is present, the encounter is not recorded for that visit to ensure privacy and compliance. The full process of recruitment is visualized in Figure 1.

Eligibility, consent, and encounter-level participation are tracked in a recruitment log to support participant flow reporting and feasibility monitoring.

### Recruitment Process

A team of 5 trained nonclinical research staff manages all recruitment and data collection activities. The standardized recruitment process is as follows:

- Daily schedule review: research staff review participating clinicians’ schedules to identify eligible new patients and previously consented patients returning for follow-up appointments.
- Low-pressure approach: research staff approach eligible patients in the exam room using a standardized, institutional review board (IRB)–approved oral script (Multimedia Appendix 1). The script uses clear, lay language to explain the study’s purpose, emphasizing that participation is entirely voluntary, privacy will be protected, and the recording can be paused or stopped at any time without affecting their care.
- Consent and confirmation: new patients provide informed consent on a tablet. For returning patients who have previously consented, the staff obtains verbal confirmation that they are still comfortable being recorded. Full written reconsent is only required if there are protocol changes.
- Declinations: patients may decline recording for any appointment. Reasons for refusal are logged by staff to monitor for potential selection bias. Patients who decline are not re-approached the same day but may be invited to participate again at future visits unless they issue a blanket refusal for all future participation.

Following recruitment and consent, the multimodal encounter capture workflow is implemented as described in the “System Components and Workflow” section.

### System Components and Workflow

This section describes the operational workflow used to deliver the multimodal encounter capture system, as well as the standardized procedures used to collect each data stream.

#### Video Recordings

Encounters are recorded using a 360° video, 2D monocular camera (eg, Insta360×4). The camera is configured at 5.7K resolution and 30 frames per second (fps; ~100 Mbps) to balance image quality with stable performance during longer outpatient visits (eg, reduced risk of overheating). We selected a consumer-grade 360° camera to enable wide-angle capture while keeping the system low-cost and replicable across clinical settings. Recording specifications and quality control criteria used to classify recordings as complete or usable are summarized in Table 1.

**Table 1.** Recording specifications and criteria for recordings.

Domain	Specification	Quality control criterion
Video	360° video; 5.7K resolution; 30 fps; approximately 100 Mbps	File opens/plays; no corruption; continuous capture across encounter (no premature stop)
Audio	Stereo dual-channel at 48 kHz; wind reduction disabled; planned downsampling to 16 kHz	Both channels present; speech audible; no sustained dropout or severe clipping preventing analysis
Placement	Concealed tabletop placement; ≥2.5 ft distance	Patient–clinician interaction area visible for most of visit
Seam avoidance	Avoid 360° seam or stitch zone	No seam artifacts affecting the interaction zone
Occlusion	Positioning to avoid obstruction by room objects	No prolonged occlusion that prevents observation or feature extraction
Duration	Recording intended to cover full encounter	Recording duration consistent with visit length; no early termination attributable to device failure
File integrity and upload	Same-day upload to secure server and upload verification	Successful upload verification; retrievable file stored on server

The camera is positioned discreetly on a tabletop stand and aligned so participants remain at an optimal distance (≥2.5 ft) and away from the camera’s stitch or seam line, where visual artifacts are most common. Audio is captured in stereo dual-channel at 48 kHz using the camera’s “stereo” mode to preserve both patient and clinician voices, with planned downsampling to 16 kHz for speech analytics. “Wind reduction” is disabled to avoid distortion of vocal frequencies relevant for acoustic analysis. To support continuous operation across clinic sessions, batteries are rotated between encounters. Participants are informed that they may pause, redirect, or stop the recording at any time. The 5.7K or 30 fps setting allows for at least 135 minutes of recording time in the latest 360°, 2D monocular camera, sufficient for typical outpatient visits.

**Figure 2.**
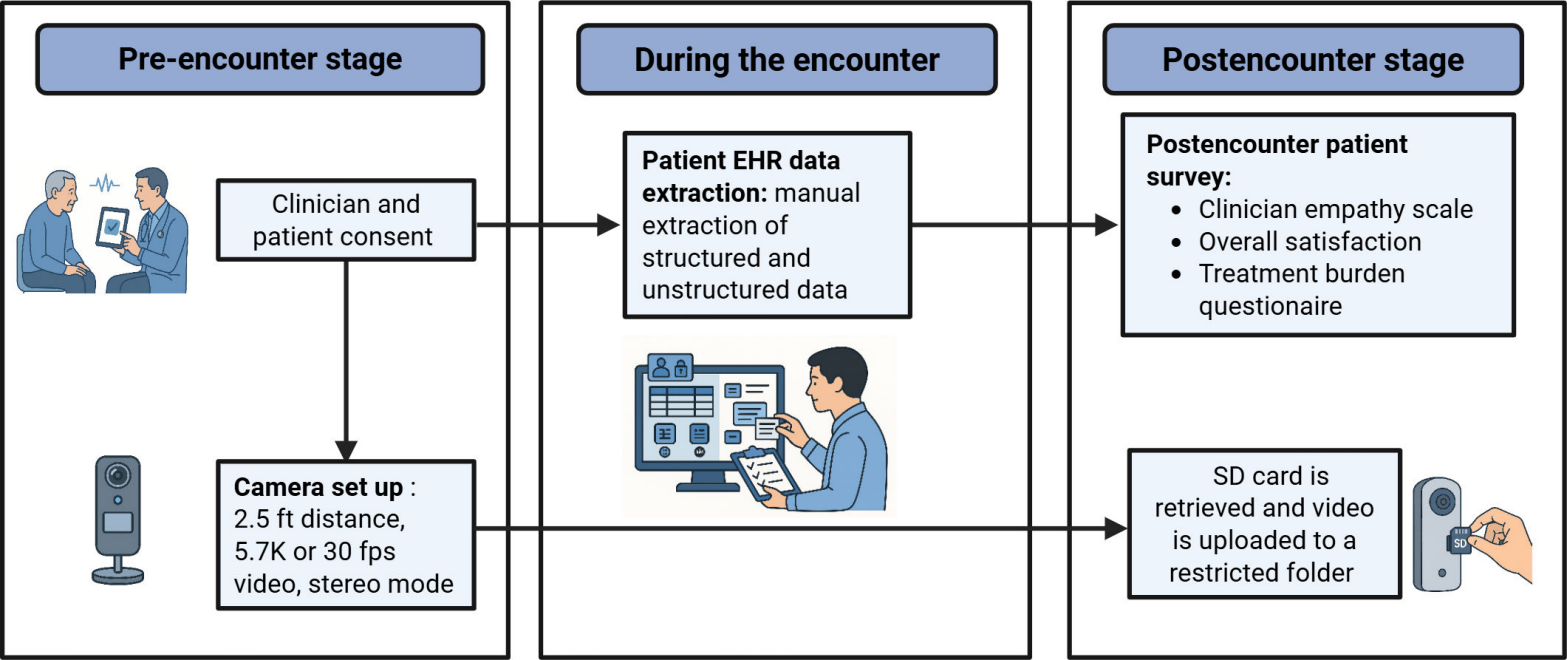
Camera setup: Multimodal encounter capture. The system integrates 3 stages: pre-encounter stage (clinician and patient consent and camera setup), during the encounter (video or audio recording and manual electronic health record [EHR] data extraction), and postencounter (patient survey completion and secure video upload).

A standardized workflow is followed daily to ensure data integrity and security. At the end of each clinic session, the SD card is retrieved, and original camera files (INSV) are transferred to an institutional workstation. Files are then converted to MP4 using the manufacturer’s software (when applicable) and uploaded the same day to a restricted-access, Health Insurance Portability and Accountability Act (HIPAA)–compliant institutional server. All files are renamed using a harmonized participant ID and encounter date. After upload verification, raw files are deleted from the SD card and any intermediate workstations. The daily workflow is illustrated in Figure 2. These standardized procedures ensure that recording, transfer, and storage processes are consistent across encounters and reproducible in other clinical settings.

#### Postencounter Patient Surveys

Immediately following the clinical encounter, patients are invited to complete a brief postvisit survey administered on a tablet. The survey includes the following validated instruments: (1) the Consultation and Relational Empathy (CARE) measure to assess patient-perceived clinician empathy and overall satisfaction, (2) the Unhurried Conversations Assessment Tool (UCAT) to assess perceived visit pace (unhurried vs rushed), and (3) the Treatment Burden Questionnaire plus Digital (TBQ+D) to assess overall treatment burden including burden related to digital care (Multimedia Appendix 2) [8-12]. It also includes the following 2 open-ended questions designed to elicit qualitative feedback:

- “Tell us how, if at all, did your visit with an endocrinology clinician help you?”
- “If you had a magic wand and you could change one thing about your visit today, what would that be?”

CARE and UCAT are scored according to published guidance, with higher scores indicating greater perceived empathy and more unhurried communication, respectively. TBQ+D is scored by summing item responses to generate a total burden score (higher scores indicate greater burden). TBQ+D is administered to all consented patients after each recorded encounter.

#### Handling of Missing Survey Data

Surveys are classified as complete, partial, or missing. For feasibility reporting, a survey is considered complete when the CARE and UCAT instruments are completed and at least 2 key TBQ+D items (financial burden and arranging medical appointments) are answered. This operational definition ensures that surveys classified as complete include both encounter-experience measures and key treatment workload items. Completion of the full TBQ+D is not required for the survey to be counted as complete. All submitted surveys are retained, including those with incomplete responses.

Instrument scores are calculated using available responses; no imputation or prorating of missing items is performed. When items are missing, scores reflect only the responses provided, and the extent of missingness will be summarized descriptively.

If a survey is not completed in clinic, study staff send up to 3 reminders via email and/or the patient portal within two weeks of the encounter. Surveys completed after reminders are retained and considered completed for feasibility analyses. Completion mode (in-clinic vs remote) and timing are recorded to allow descriptive assessment of response patterns.

#### EHR Data Extraction

Following a recorded encounter, trained study staff perform a manual extraction of structured (demographic and clinical variables) and unstructured (clinical notes and patient portal messages) data from the patient’s EHR. Structured data include age, sex, race or ethnicity, clinical diagnoses, visit type (eg, new or follow-up), and current medications.

### Pilot Phase and Iterative Refinement

The system is piloted over 3 weeks, with 30 recorded encounters (~10 per wk) to identify and resolve logistical issues related to camera placement (eg, ensuring the camera is unobtrusive), workflow disruption (eg, additional steps or delays during rooming), and recording quality (eg, interruptions or file-transfer failures). Pilot feedback is reviewed weekly, and the recruitment script, equipment handling, and data processing steps are refined before initiating full-scale recruitment. Early workflow and technical failures are documented and inform refinements to standardized camera placement, battery management, and file verification procedures prior to full-scale recruitment. Recorded encounters from the pilot phase can be included in the final dataset.

The system is refined based on three design constraints to ensure the final system is scalable and replicable: (1) comprehensive video capture: the system must provide 360-degree video coverage without requiring a dedicated camera operator in the room; (2) high-quality audio: the system must capture dual-channel audio at a sample rate sufficient for automated speech recognition and diarization (≥16 kHz); and (3) minimal clinic disruption: equipment setup and recording initiation must fit within the standard patient rooming process, without extending visit times.

### Data Integration and Management

#### Triangulation and Harmonization

The study is designed to capture three components: (1) 360° video-recorded clinical interactions, (2) post-encounter patient surveys, and (3) structured and unstructured data extracts from the EHR. These sources are harmonized using an identification schema and documented in a central tracking log.

For clinicians, the log tracks their professional role, consent status, and completion of the demographic survey. For patients, it records demographic data (age, sex, and race or ethnicity), eligibility status with documented reasons for ineligibility, guest presence and consent status, recording success, video upload success, postvisit survey completion status (complete, partial, or none), and any challenges noted by the research staff (eg, equipment malfunction and patient discomfort). Recording failure modes are logged in the encounter tracking log.

Each entry is indexed by the unique participant ID, connecting all 3 data streams. All files follow a harmonized naming convention based on the participant ID and encounter date to allow reliable data merging and adherence to the findable, accessible, interoperable, and reusable data principles, maximizing the use of the collected data for future research. Table 2 summarizes the study’s data sources and how multimodal data are formatted, linked, and stored securely. This structured approach ensures reliable data merging and supports complex, temporally aligned multimodal analyses.

**Table 2.** Multimodal data streams, key variables, and linkage schema.

Data source	Modality	Key variables or format	Linkage key	Storage platform
Camera	Video	360° video, 5.7K or 30 fps, H.265 codec, and .mp4 format	PATIENT_ID_ENCOUNTER_DATE.mp4	Secure institutional server
Camera	Audio	Dual-channel, 48 kHz WAV, and down sampled to 16 kHz	PATIENT_ID_ENCOUNTER_DATE.wav	Secure institutional server
Survey	Patient-reported	Empathy, satisfaction, burden (Likert scales), and open-ended text	PATIENT_ID, ENCOUNTER_DATE	REDCap^a^ (Vanderbilt University)
EHR^b^	Structured and unstructured information	Demographics, ICD-10^c^ codes, medications, and laboratory values	PATIENT_ID, ENCOUNTER_DATE	REDCap (via manual extraction)

a REDCap: Research Electronic Data Capture.

b EHR: electronic health record.

c ICD-10: *International Classification of Diseases, Tenth Revision.*

#### Storage and Security

Video and audio recordings are labeled with study-specific IDs and uploaded daily to a secure institutional server with institutional authentication and role-based access controls. Following upload verification, raw files are deleted from the camera’s SD card and any intermediate workstations. All study data are stored in accordance with HIPAA regulations and institutional data security protocols and are protected using institutional security safeguards, including encryption protections. Access to identifiable recordings is restricted to authorized study personnel using institutional authentication and role-based permissions, with all access auditable under institutional information security practices.

Survey and EHR data are managed and stored using REDCap (Vanderbilt University), a secure, web-based application designed for research data collection and management. The final analysis dataset is fully deidentified, with the unique participant ID serving as the only link between the different data modalities. Patients who withdrew consent or became ineligible during the study are excluded from the final dataset unless explicit permission is granted to retain their previously collected data.

In accordance with IRB approval and participant authorization, identifiable encounter recordings and linked study data may be retained in a secure research registry for ongoing and future IRB-approved research. Participant authorization to use and share health information is not time-limited and remains in effect unless revoked. Participants may withdraw from the study at any time, after which no additional data will be collected. Participants may also revoke authorization for future use of their information, in which case their information will be removed from repositories where feasible; however, information already distributed for research use cannot be retrieved. Secondary use, including AI or machine learning model development or training, is restricted to IRB-approved purposes and to the scope described in the consent or authorization. External sharing is not planned as part of this feasibility study; any future sharing with collaborators outside the institution would require IRB review and approval and would occur only under a formal data use agreement specifying permitted uses and access restrictions.

After data collection and integration procedures are completed, predefined feasibility and analytic outcomes are assessed as described below.

### Data Analysis

The primary outcomes of this feasibility protocol are predefined operational end points reflecting both the feasibility and acceptability of the system, including clinician and patient consent rates, recording success, survey completion, and multimodal data linkage (Table 3).

**Table 3.** Primary feasibility end points, definitions, and success thresholds.

End point	Definition (numerator/denominator)^a^	Success threshold (%)
1. Clinician consent rate	Consented clinicians/all approached clinicians	>90
2. Patient consent rate	Consented patients/all approached eligible patients	>50
3. Recording success rate	Complete or usable recordings/all consented encounters	>90
4. Survey completion rate	Completed surveys/all consented encounters	>90
5. Data linkage rate	Encounters with linked video, survey, and EHR^b^/all consented encounters	>90

a Feasibility rates are calculated as numerator divided by denominator, where the numerator represents the number of clinicians, patients, or encounters meeting the specified endpoint and the denominator represents the total number of eligible clinicians, patients, or encounters.

b EHR: electronic health record.

This protocol reports interim feasibility metrics; additional analytical findings will be reported in a subsequent publication.

#### Feasibility and Recruitment Metrics

The study’s feasibility is measured using 5 predefined end points. These end points, their definitions, and the a priori success thresholds are detailed in Table 3. Descriptive statistics (counts and proportions) are calculated for each end point using data from the recruitment tracking logs and REDCap exports.

All feasibility end points, except clinician and patient consent rates, are calculated at the encounter level, as patients may contribute multiple encounters.

#### Planned Analysis of Data Components

#### Overview

The analysis of the 3 components (video recordings, postencounter surveys, and EHR data) will be described, in a future publication, using descriptive statistics. In addition to feasibility end points (Table 3), we will generate encounter-level derived measures from audio or video, survey, and EHR data to support downstream encounter research and AI development. Key derived measures include encounter duration, speech and turn-taking metrics (eg, speaking time per speaker, number of turns, turn length, overlap or interruption frequency, and pause frequency or duration), acoustic or prosodic features (eg, fundamental frequency and intensity summaries), and selected nonverbal behavior features (eg, face presence, gaze direction, head nodding, and smile frequency). Measures sensitive to encounter length will be reported both as absolute values and normalized per minute.

Because patients may contribute multiple encounters and clinicians may contribute encounters with multiple patients, downstream analyses will account for repeated measures and clustering by clinician using mixed-effects models (eg, random intercepts for clinician and patient as appropriate) and/or cluster-robust standard errors. Analyses will also be summarized overall and stratified by visit type (new vs follow-up) and clinician role.

##### Acoustic Feature Extraction

Video recordings will be automatically transcribed and diarized using the best available models [13,14]. From the audio, key acoustic features will be extracted using the *Parselmouth* Python library (Python Software Foundation), which provides access to the Praat gold-standard algorithms [15]. These include: (1) prosodic features (fundamental frequency mean and SD, and intensity contours), (2) spectral features (formants F1-F3, related to vowel articulation, and (3) temporal features (speech rate, and pause duration and frequency). This allows for quantitative analysis of conversational dynamics such as turn-taking, speech overlap, and backchannels.

##### Visual Feature Extraction

A planned analysis pipeline using the Open Computer Vision Library (OpenCV) [16] will be used to extract nonverbal cues. This process will involve: (1) face detection using pretrained deep neural network models to locate patient and clinician faces in each frame [16,17], (2) facial landmark detection to identify key points (eg, corners of the eyes and mouth) [16,18], and (3) approximations of facial action units corresponding to specific muscle movements (eg, smiling and brow furrowing) [16,18]. Additional visual features such as smile, nod, gaze, and emotion detection will be extracted using OpenCV and Emotion-Qwen [16,19].

##### Advanced Emotion and Behavior Analysis

The dataset will be leveraged to train and validate advanced models like Emotion-Qwen [19], a state-of-the-art large multimodal model. This approach will support analysis that moves beyond simple emotion labels (eg, “happy” and “sad”) to more complex, context-aware reasoning about the affective dynamics of the conversation, such as identifying moments of empathy, confusion, or shared understanding.

##### Postencounter Surveys and EHR Data

Data from the postencounter surveys will be used to calculate the mean scores, SDs, and distribution of each scale item. Where relevant, responses will be stratified by key variables of interest, such as clinician type (eg, physician, fellow, and educator) and visit type (eg, new vs follow-up). For the subset of patients who complete the TBQ+D, total scores and item-level responses will be summarized to describe perceived treatment workload. Analysis will be performed using SAS (version 9.4; SAS Institute).

Structured data from EHRs will be described in the same manner as post-encounter survey results, whereas unstructured data (ie, clinical notes and patient portal messages) will be described in terms of length (number of words).

### Ethical Considerations

This study was approved by the Mayo Clinic IRB (number 24‐012956). All participating clinicians and patients provided informed written consent prior to any recording. Adult guests present in the exam room were asked to provide verbal consent prior to recording; encounters were not recorded if a guest declined or if a minor was present. Participants could request that recording be paused or stopped at any time and could withdraw from the study without affecting their clinical care.

Identifiable audio and 360° video recordings, along with linked survey and electronic health record data, are stored on secure institutional systems. Access to identifiable recordings is restricted to authorized study personnel and is granted only after an individual is added to the IRB-approved study personnel list and receives institutionally approved access. Access provisioning and revocation are managed through institutional information security processes. All access to identifiable data is logged and auditable under institutional security monitoring practices.

Study data, including identifiable encounter recordings, may be retained in a secure research registry without a predefined retention end date, consistent with IRB approval and participant authorization. If a participant withdraws from the study, no additional data are collected; however, data collected prior to withdrawal may be retained and used for research purposes unless the participant explicitly revokes authorization for future use, in accordance with institutional policy.

Secondary use of the data, including use for AI or machine learning research, is permitted only under IRB-approved protocols and within the scope described in the informed consent and authorization. External data sharing is not planned as part of this feasibility study; any future sharing with collaborators outside the institution would require additional IRB review and approval and would occur only under a formal data use agreement specifying permitted uses and access restrictions. No compensation is provided for participation.

## Results

The protocol was implemented in the outpatient clinic of the Division of Endocrinology at Mayo Clinic in Rochester, Minnesota, an academic specialty clinic. We selected the Insta360×4 camera because it met all required specifications, including 5.7K resolution at 30 frames per second (~100 Mbps) with dual-channel 48 kHz audio, for a longitudinal 360° 2D encounter capture system. Video files were recorded in INSV format and converted to MP4 using the Insta360 software (Insta360, Shenzhen, China). Pilot-phase recordings meeting the predefined quality control criteria (Table 1) were eligible for inclusion in the final dataset.

Recruitment and encounter recording began in January 2025. By August 2025, 35 of 36 (97%) eligible clinicians and 212 of 281 (75%) approached patients had consented. Of the consented encounters, 162 (76%) resulted in a complete or usable 360° video recording, and the postvisit surveys were completed for 204 of 212 (96%) consented encounters, reflecting 1 survey per encounter. At the time of the interim analysis, 150 of 212 (71%) consented encounters had successfully linked video, survey, and EHR data. Data collection is ongoing as of December 2025, and additional analyses of the multimodal dataset will be reported in subsequent publications.

## Discussion

### Principal Results

This protocol describes a scalable framework for capturing rich, multimodal, longitudinal, real-world clinical encounters in chronic disease care. The primary objective of this study is to assess feasibility, determined by achieving predefined thresholds for recruitment, data capture, and data linkage. Interim feasibility metrics include 97% clinician consent, 75% patient consent, and 96% postvisit survey completion across consented encounters, reflecting 1 survey per encounter. Although recording success (complete or usable 360° recordings) was 76% of consented encounters to date, common reasons for incomplete or unusable recordings included workflow interruptions (eg, inconsistent camera placement leading to occlusion or seam artifacts, visit-length variability, and battery depletion) and intermittent device malfunction (eg, unexpected shutoffs requiring servicing). These workflow and equipment issues led to protocol refinements, including standardized camera placement guidance to minimize occlusion and seam artifacts, a fixed battery rotation schedule aligned with anticipated visit duration, and same-day verification of file integrity following each encounter. Similarly, linkage across video, survey, and EHR data streams was lower during early implementation primarily because some encounters lacked a complete recording, which is required for multimodal linkage. Improvements to recording workflows and quality control procedures described above are expected to improve linkage rates in subsequent phases.

These implementation metrics describe workflow integration in an outpatient specialty clinic and inform subsequent planned analyses. The collected corpus is designed to support downstream person-centered AI and encounter research by enabling multimodal analysis of real-world clinical interactions.

### Comparison With Prior Work

There have been similar efforts to capture multimodal information from natural conversations, but few have focused on real-world health care encounters. To the best of our knowledge, prior studies in health care have been limited to audio-only transcript analysis, such as applying natural language processing to primary care conversations [6], or video capture in simulated training settings rather than routine clinical care [20]. Outside of health care, multimodal datasets such as the CANDOR corpus have advanced the study of natural conversation, but these do not address the ethical, logistical, and workflow challenges unique to clinical environments, such as HIPAA compliance and the need to avoid disrupting care delivery [7].

This protocol advances the field by embedding multimodal capture directly into routine specialty care, an approach not addressed in previous studies. The iterative piloting with clinical staff illustrates stakeholder engagement, ensuring the system fits existing workflows and minimizes disruption, a principle emphasized in implementation science. The harmonized data linkage strategy enhances feasibility and replicability by reliably integrating video, survey, and EHR data. Finally, the use of low-cost, general-purpose hardware increases scalability and democratizes access to multimodal encounter research, including resource-limited settings.

### Next Steps and Future Directions

The immediate next steps involve analyzing the rich dataset that this protocol enables, with initial work focused on correlating patient-reported outcomes with observable communication dynamics. For example, verbal and nonverbal behaviors such as speaking time, turn-taking, and gaze can be examined in relation to patient-reported empathy, satisfaction, and treatment burden. Planned analyses will examine conversational moments characterized by empathy, attentiveness, and shared decision-making in relation to patient-reported empathy, satisfaction, visit pace, and treatment burden. Linking observed communication behaviors to patient-reported outcomes will be explored to characterize patterns associated with higher or lower perceived treatment burden, particularly in relation to navigating services and managing costs.

Beyond these initial projects, the dataset presents significant opportunities for advanced AI research, though not without challenges. Processing each modality introduces unique complexities: audio data requires robust speaker diarization and transcription models tuned for clinical conversations; video data presents challenges in data volume, storage, and the computational cost of extracting nonverbal cues like facial expressions and gestures; and integrating these streams with EHR and survey data requires sophisticated data fusion techniques to handle issues such as missing data and ensure temporal alignment.

Furthermore, while this protocol was implemented in endocrinology, the framework is designed for broader applicability. Future work will involve deploying this system in other clinical settings, such as oncology, where complex treatment decisions and longitudinal patient relationships are also central to care. In oncology, integrating encounter data with genomic, imaging, and clinical data may inform future development of AI models to support shared decision-making and personalize patient communication, augmenting rather than replacing clinical judgment. By systematically addressing these next steps, this work can help build the foundational datasets needed to move beyond EHR-based models and develop AI that truly understands the human context of clinical care.

Finally, it is important to address the question of scalability. While this protocol describes a replicable framework, the long-term vision is not to place a camera in every exam room for every encounter, which may not be practical or desirable. Instead, this foundational research is a crucial step toward the development of more integrated and less obtrusive ambient intelligence technologies. These future systems, embedded seamlessly into the clinical environment, could capture the essential multimodal data of an encounter without the need for standalone recording devices. The insights gained from this study, regarding consent workflows, patient and clinician acceptance, and ethical safeguards, will be invaluable for informing the design of next-generation ambient systems that can capture encounter data at scale while maintaining the trust and privacy essential to the patient-clinician relationship. By systematically addressing these next steps, this work can help build the foundational datasets needed to move beyond EHR-based models and develop AI that truly understands the human context of clinical care.

### Limitations

This framework faces potential challenges, and the protocol includes specific strategies to mitigate them. One concern is selection bias, where patients who consent to participate may differ systematically from those who do not—for example, by being more comfortable with technology or having higher trust in the health care system. To address this, the protocol includes logging reasons for refusal, using a low-pressure consent script to build trust, and conducting a comparative analysis of key variables between participants and non-participants to quantify any observed bias. In particular, we will examine whether consent rates vary by visit type (new vs follow-up) and clinician role (eg, physician, fellow, educator, and advanced practice provider), and we will compare available characteristics of consenting versus eligible nonconsenting patients to assess representativeness (eg, age, sex, and race or ethnicity when available). Another potential issue is the Hawthorne effect [21], where the presence of a recording device may alter the natural behavior of participants. The study’s longitudinal design serves as the primary mitigation, as reactivity is expected to diminish over time as participants become habituated to the recording process. The use of a small, unobtrusive camera is also intended to minimize the salience of the observation.

The protocol also proactively addresses potential challenges related to the technical reliability of consumer-grade hardware. Because high-resolution 360° video can overheat cameras and exhaust storage, our protocol includes capping resolution at 5.7 K, rotating batteries on a fixed schedule, and formatting SD cards daily. Finally, as a single-site study in a specialized clinic, the findings may not be immediately generalizable. This study is intended as a foundational step, and if feasibility is demonstrated, future multisite trials will be essential to establish broader applicability.

### Conclusions

This protocol describes a rigorous, transparent, and replicable method for capturing the multimodal dynamics of clinical encounters in chronic disease care. By detailing operational workflows, technical specifications, feasibility end points (including clinician and patient consent, recording success, survey completion, and data linkage), and mitigation strategies, it offers a practical template for other research teams. These end points use prespecified operational definitions and quality checks, including what constitutes a recorded encounter, a complete or usable 360° recording (full-length capture with acceptable audio or video quality and no critical occlusion or file corruption), and a linked encounter (successful matching of video, survey, and EHR data using the harmonized participant ID and encounter date). Demonstrating the feasibility of this longitudinal approach establishes a resource for developing next-generation, person-centered AI tools that can understand and respond to patients’ lived experiences. This work represents a foundational step toward building AI that can appreciate both the “biology” and the “biography” of a patient, helping make health care more effective, equitable, and humane.

## Supplementary material

10.2196/84688 Multimedia Appendix 1 Recruitment script.

10.2196/84688 Multimedia Appendix 2 Postencounter patient survey.

## Abbreviations

AI: artificial intelligence
CARE: Consultation and Relational Empathy
EHR: electronic health record
HIPAA: Health Insurance Portability and Accountability Act
IRB: institutional review board
TBQ: Treatment Burden Questionnaire
TBQ+D: Treatment Burden Questionnaire plus Digital
UCAT: Unhurried Conversations Assessment Tool
