# Supplementary material for: Longitudinal and Multimodal Recording System to Capture Real-World Patient-Clinician Conversations for AI and Encounter Research: Protocol for an Observational Study
Source: JMIR Res Protoc. 2026 Mar 24;15:e84688. doi: 10.2196/84688 (PMC13012220; doi:10.2196/84688)
Supplement: Multimedia Appendix 1 [file resprot-v15-e84688-s001.docx]

**Multimedia Appendix 1. Recruitment script**

*Hello, my name is [NAME]. I am part of the research team here in the [Department/Clinic]. We are conducting a research study to better understand how patients and clinicians communicate and interact during visits. Participation is voluntary. If you are interested, you would sign a consent form, we would place a small recording device in the room during your appointment, and after your visit you would complete a short survey. The device records video and audio of the visit, but most people forget about it after a few minutes. You can pause or stop the recording at any time, and your care will not be affected if you choose not to participate. Would you like to hear more about the study?*
