# Supplementary material for: Longitudinal and Multimodal Recording System to Capture Real-World Patient-Clinician Conversations for AI and Encounter Research: Protocol for an Observational Study
Source: JMIR Res Protoc. 2026 Mar 24;15:e84688. doi: 10.2196/84688 (PMC13012220; doi:10.2196/84688)

**Multimedia Appendix 2. Post-Encounter Patient Survey**

**DID WE CARE WELL TODAY?**

A research study

Thank you for helping us with this study. We are asking you about your visit today.

Your responses are completely confidential and voluntary, will be used for research only, and will not be shared with anyone outside of the research team.

**What is the main reason for visiting Endocrinology at Mayo Clinic today?** *(Select more than one if needed)* 
☐ Thyroid Nodule 
☐ Thyroid Cancer 
☐ Other Thyroid Condition 
☐ Diabetes 
☐ Weight Management 
☐ Osteoporosis or Osteopenia 
☐ Adrenal Disorders 
☐ Pituitary Disorders 
☐ Calcium or Parathyroid Concerns 
☐ Testosterone Concern 
☐ Polycystic Ovary Syndrome (PCOS) 
☐ Other (please specify): __________________________ 
☐ Prefer Not to Disclose

**Is this your first visit to Endocrinology at Mayo Clinic for this problem?**

☐ Yes 
☐ No

☐ Not sure

☐ Prefer not to answer

**Kindly mark the box next to each statement to indicate how was your endocrinology visit was today.**

| **How good was the clinician at...** | **Poor** | **Fair** | **Good** | **Very Good** | **Excellent** | **Does not apply** |
| --- | --- | --- | --- | --- | --- | --- |
| **1) Making you feel at ease**  (introducing themselves, being friendly, respectful, and warm towards you; not cold or abrupt) | 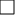 | 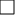 | 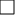 | 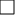 | 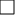 |  |
| **2) Letting you tell your "story"**  (giving you time to describe your condition in your own words; not interrupting, rushing or diverting you) |  |  |  |  |  |  |
| **3) Really listening**  (paying close attention to what you were saying, not looking at the notes or computer as you were talking) |  |  |  |  |  |  |
| **4) Being interested in you as a whole person** (asking/knowing relevant details about your life, your situation; not treating you as "just a number") |  |  |  |  |  |  |
| **5) Fully understanding your concerns** (communicating that they had accurately understood your concerns and anxieties; not overlooking or dismissing anything) |  |  |  |  |  |  |
| **6) Showing care and compassion**  (seeming genuinely concerned, connecting with you on a human level; not being indifferent or "detached") |  |  |  |  |  |  |
| **7) Being positive**  (having a positive approach and a positive attitude; being honest but not negative about your problems) |  |  |  |  |  |  |
| **8) Explaining things clearly**  (fully answering your questions; providing clear and adequate information; not being vague) |  |  |  |  |  |  |
| **9) Helping you to take control**  (exploring with you what you can do to improve your health yourself; encouraging rather than "lecturing" you) |  |  |  |  |  |  |
| **10) Making a plan of action with you**  (discussing the options, involving you in decisions as much as you want to be involved; not ignoring your views) |  |  |  |  |  |  |

**Kindly mark the box next to each statement to indicate how your endocrinology visit was today.**

|  | **Not very** | **Minimally** | **Unsure** | **Somewhat** | **Very** |
| --- | --- | --- | --- | --- | --- |
| **11)** How **helpful** was your visit with the clinician? |  |  |  |  |  |
| **12)** How **rushed** was your visit with the clinician today? |  |  |  |  |  |
|  | **Not at all** | **Probably not** | **Unsure** | **Probably yes** | **Defnitely** |
| **13)** Likelihood of you recommending this clinician to others |  |  |  |  |  |

**Tell us how, if at all, did your visit with an endocrinology clinician help you?**


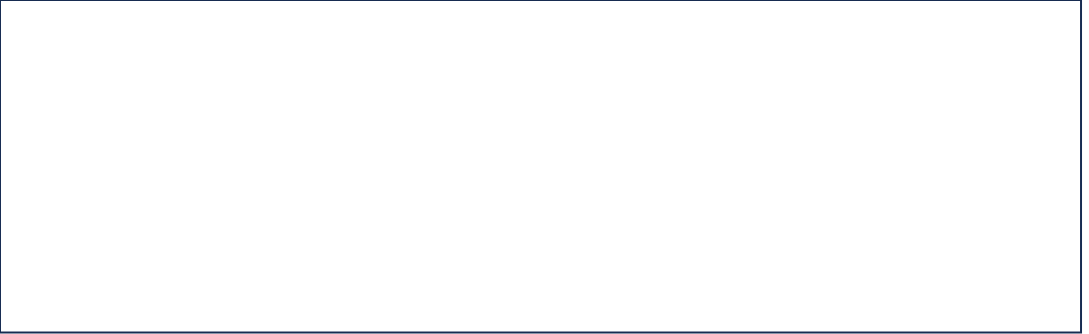


**Now take a moment and consider everything you have to do to take care of your health. Please rate the burden or problem associated with each of the following items using the following scale:**

**How would you rate the problems related to:**

Arranging medical appointments (doctor visits, lab tests and other exams) and reorganizing your schedule around these appointments

| Does not apply | Not a Problem  0 | 1 | 2 | 3 | 4 | 5 | 6 | 7 | 8 | 9 | Big Problem 10 |
| --- | --- | --- | --- | --- | --- | --- | --- | --- | --- | --- | --- |
| □ | □ | □ | □ | □ | □ | □ | □ | □ | □ | □ | □ |

The financial burden associated with your healthcare (for example: out of pocket expenses or expenses not covered by insurance)?

| Does not apply | Not a Problem  0 | 1 | 2 | 3 | 4 | 5 | 6 | 7 | 8 | 9 | Big Problem 10 |
| --- | --- | --- | --- | --- | --- | --- | --- | --- | --- | --- | --- |
| □ | □ | □ | □ | □ | □ | □ | □ | □ | □ | □ | □ |

**If you had a magic wand and you could change one thing about your visit today, what would that be?**


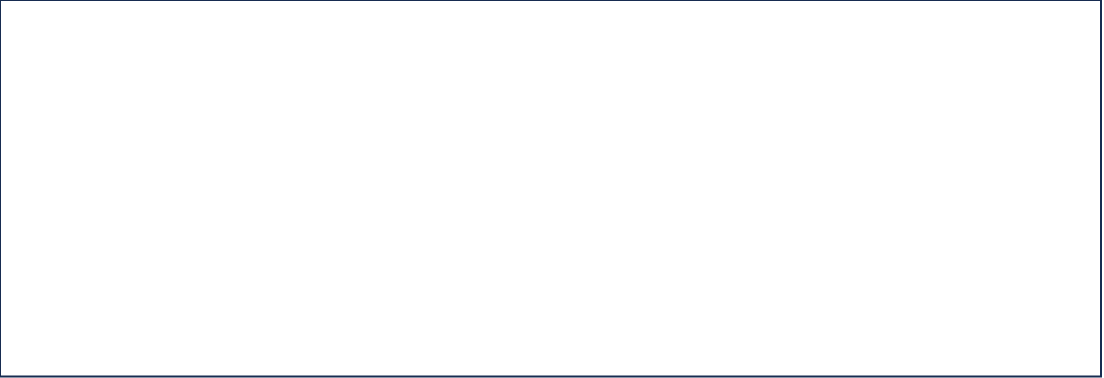

Supplement: Multimedia Appendix 2 [file resprot-v15-e84688-s002.docx]
